# HLA-Cw*0102-restricted HIV-1 p24 epitope variants can modulate the binding of the inhibitory KIR2DL2 receptor and primary NK cell function

**DOI:** 10.1186/1742-4690-9-S2-P177

**Published:** 2012-09-13

**Authors:** C Koerner, L Fadda, S Kumar, N van Teijlingen, A Piechocka-Trocha, M Carrington, M Altfeld

**Affiliations:** 1 Ragon Institute of MGH, MIT and Harvard, Charlestown, MA, USA; 2 National Cancer Institute at Frederick, Frederick, MD, USA

## Background

Recently, it was shown that Natural Killer (NK) cell-mediated immune pressure can result in the selection of HIV-1 escape mutations contributing to accumulating evidence suggesting that NK cells play an important role in the control of HIV-1 infection. Selection of HLA class I-presented HIV-1 epitopes that allow for engagement of inhibitory killer cell Ig-like receptors (KIRs) might serve as a potential mechanism for NK cell escape. We therefore investigated the consequences of sequence variations within HLA-Cw*0102-restricted epitopes on the interaction with KIR2DL2 using a large panel of HIV-1 p24 Gag peptides.

## Methods

A total of 217 decameric peptides spanning HIV-1 p24 Gag and overlapping by 9aa were screened for HLA-Cw*0102 stabilization by co-incubation with Cw*0102(+) TAP-deficient T2 cells using a flow cytometry-based assay. KIR2DL2 binding was assessed using KIR2DL2-Fc. Function of KIR2DL2(+) NK cells was flow cytometrically analyzed by measuring degranulation of primary NK cells after co-incubation with peptide-pulsed T2 cells.

## Results

We identified 11 peptides stabilizing HLA-Cw*0102 on the surface of T2 cells. However, only one peptide (p24 Gag209-218) also allowed for binding of KIR2DL2. Notably, functional analysis showed significant inhibition of KIR2DL2(+) NK cell function in the presence of p24 Gag209-218-pulsed T2 cells, while degranulation of KIR2DL2(-) NK cells was not affected. Moreover, we demonstrated that sequence variations in position 7 of this epitope observed frequently in naturally occurring HIV-1 sequences can modulate binding to KIR2DL2.

## Conclusion

Our results show that variations in HIV-1 peptides presented by HLA can modulate target cell recognition by NK cells. Understanding the mechanisms that determine NK cell-mediated recognition of HIV-1-infected cells will be a critical step for harnessing NK cell immunity for vaccine design, in particular given the recent discovery of virus-specific memory NK cells in mice (Paust et al., Nat Imm 2009). This study was supported by the NIH, Ragon Institute and HU CFAR.

